# K−12 teachers' stress and burnout during the COVID-19 pandemic: A systematic review

**DOI:** 10.3389/fpsyg.2022.920326

**Published:** 2022-09-02

**Authors:** Andrea Westphal, Eva Kalinowski, Clara Josepha Hoferichter, Miriam Vock

**Affiliations:** ^1^Interdisciplinary Research on Teaching, Learning and School Development, University of Greifswald, Greifswald, Germany; ^2^Empirical Research on Instruction and Intervention, University of Potsdam, Potsdam, Germany

**Keywords:** burnout, stress, COVID-19, pandemic, K−12 teachers, remote teaching

## Abstract

We present the first systematic literature review on stress and burnout in K−12 teachers during the COVID-19 pandemic. Based on a systematic literature search, we identified 17 studies that included 9,874 K−12 teachers from around the world. These studies showed some indication that burnout did increase during the COVID-19 pandemic. There were, however, almost no differences in the levels of stress and burnout experienced by K−12 teachers compared to individuals employed in other occupational fields. School principals' leadership styles emerged as an organizational characteristic that is highly relevant for K−12 teachers' levels of stress and burnout. Individual teacher characteristics associated with burnout were K−12 teachers' personality, self-efficacy in online teaching, and perceived vulnerability to COVID-19. In order to reduce stress, there was an indication that stress-management training in combination with training in technology use for teaching may be superior to stress-management training alone. Future research needs to adopt more longitudinal designs and examine the interplay between individual and organizational characteristics in the development of teacher stress and burnout during the COVID-19 pandemic and beyond.

## Introduction

Between spring 2020 and summer 2021, teachers and students around the world experienced school closures as a result of the COVID-19 pandemic. Although school closures are not unknown historically—occurring, for instance, during the polio pandemic in 1916 (Meyers and Thomasson, 2021) and during the influenza pandemic in 2009 (Jackson et al., 2014)—the duration and global reach of school closures during this pandemic was historically unique. At the peak of the pandemic, about 1.5 billion students were affected by school closures (UNESCO, 2021). Empirical research has focused on the considerable consequences the COVID-19 pandemic has had for students' wellbeing (Asbury et al., 2020) and achievement, especially for students from families with low socioeconomic statuses (Hammerstein et al., 2021), thus widening existing social disparities (e.g., Westphal et al., 2016). Less attention has been paid to the question of how teachers have experienced the COVID-19 pandemic, the related school closures, and the required shift to online teaching (Kim and Asbury, 2020). We aim to shed light on the struggles and strains that COVID-19 and the accompanying school closures posed for K−12 teachers, i.e., for teachers teaching in kindergarten (K) or in the first through the 12th grade (1–12). To achieve this, the present review synthesizes quantitative research on K−12 teachers' stress and burnout during the COVID-19 pandemic.

### Teacher stress and burnout

Teacher stress is a potential undesirable consequence of the COVID-19 school closures (UNESCO, 2021), emerging from issues such as uncertainties about the duration of school closures or teachers' lack of experience with remote teaching (e.g., Kim and Asbury, 2020). The question of how to tackle teacher stress and prevent teachers from leaving their profession as a result of burnout is not new to researchers, teacher educators, and stakeholders in educational policy (Abel and Sewell, 1999; OECD, 2020a).

Appraisal-based approaches to stress, such as the transactional model of stress and coping (Lazarus and Folkman, 1984; Chang, 2009), propose that teacher stress results from a teacher's perception or appraisal of an event or environment as being threatening, harmful, or as entailing stressors that exceed their coping resources. Repeated or prolonged exposure to stressors and inadequate coping strategies may make the symptoms of burnout more likely (Lazarus, 1999). Burnout is defined as a multidimensional “syndrome of emotional exhaustion, depersonalization, and reduced personal accomplishment” (Maslach et al., 1996, p. 4). Emotional exhaustion is seen as the core element of burnout (Maslach et al., 2001). It comprises physical fatigue and emotional depletion in the workplace and is the symptom most commonly reported by individuals who suffer from burnout (Maslach et al., 2001). Depersonalization is characterized by an indifference toward others in the working context, i.e., students and colleagues in a school. Reduced personal accomplishment describes the process of becoming less efficient in finishing important tasks at work. The Maslach Burnout Inventory, developed by Maslach and Jackson (1981), allows researchers to assess these three dimensions of burnout.

### Drivers of teacher stress and burnout

Up until the 1970's, workload, time pressure, and physical strain were seen as the essential drivers of distressing experiences in the workplace (Karasek, 1979). Building on this view, the demand-control model (Karasek, 1979) explained stress at work as resulting from a combination of high job demand and low job control. Demerouti et al. (2001) underlined the important role of other resources beyond job control, which they framed as physical, organizational, social, and psychological factors in the workplace that may either facilitate the achievement of job goals, including individual growth and development, or ameliorate the detrimental consequences of job demands (Demerouti et al., 2001). The job-demands-resources model (Demerouti et al., 2001) integrates these definitions, suggesting that job demands increase the risk of burnout, while job resources can have both directly positive effects, decreasing the risk of burnout, as well as ameliorating effects that lessen the negative consequences of job demands. The job-demands-resources model stimulated research on the questions of “‘what' causes burnout?” and “‘who' gets burned out?” (Chang, 2009, p. 200).

Empirical research on the question of what causes teacher burnout has shown that discipline problems (meta-analysis by Aloe et al., 2014; Skaalvik and Skaalvik, 2017), low student motivation (e.g., Friedman, 1995; Skaalvik and Skaalvik, 2016, 2017), and a dissonance between teacher and student values (e.g., Skaalvik and Skaalvik, 2017) all play a crucial role in teacher burnout. In addition to this, time pressure or work overload may contribute to the development of teacher burnout (e.g., Goddard et al., 2006; Betoret and Artiga, 2010; Skaalvik and Skaalvik, 2010, 2011, 2017; Fernet et al., 2012). In terms of the question of “who gets burned out,” meta-analyses has shown that teachers' personality traits—especially a high level of emotional stability and extraversion (Cramer and Binder, 2015; Kim et al., 2019)—can make teachers less susceptible to burnout. In addition, teachers with higher self-efficacy in classroom management are less likely to be affected by burnout (meta-analysis by Aloe et al., 2014). Research findings are, however, inconsistent on the question of whether teachers' age, gender, and/or teaching experience make them more vulnerable to burnout (e.g., review by Chang, 2009; Mota et al., 2021).

Appraisal-based approaches to stress and burnout illustrate the interplay between personal characteristics and job characteristics. Consequently, more recent studies on burnout have shifted their attention to the question of “‘who' gets burned out in ‘which' situations?” (Chang, 2009, p. 201). Most evidence on this question has been gathered in the context of the interplay between classroom disturbances, teacher characteristics, and burnout. For instance, a study by Dicke et al. (2015b) showed that teachers with higher levels of classroom-management self-efficacy were less susceptible to emotional exhaustion when teaching in schools with a higher level of classroom disturbances than those teachers who reported lower levels of self-efficacy (see also Evers et al., 2004).

### Challenges for teachers during the COVID-19 pandemic

The COVID-19 pandemic demanded profound changes in everyday teaching (Reimers and Schleicher, 2020) “likely to be cognitively and emotionally taxing for teachers” (Kim and Asbury, 2020, p. 1,063). Teachers have had to quickly adapt their lessons to remote teaching, which may have been a considerable challenge for many. In 2018, i.e., prior to the pandemic, across all countries participating in the PISA study, one third of 15-year-old students were attending schools whose principals indicated that most of their teachers did not possess the relevant pedagogical and technical skills to utilize digital devices in their lessons (OECD, 2020b). Teachers' digital and pedagogical skills (as reported by school principals) varied substantially within countries, with socio-economically advantaged schools having considerably higher digital and pedagogical teacher skills than socio-economically disadvantaged schools (OECD, 2020b). What might have further complicated remote teaching is that a not inconsiderable number of students only had restricted home access to the internet and to computers (OECD, 2022a,b).

In addition to these issues, many teachers have had to face competing responsibilities when preparing their online lessons while caring for their own children at home, which often resulted in increased parenting stress and work overload (Hong et al., 2021). Given the high incidence of COVID-19 in many countries, we can also assume that a number of teachers have also had to take care of family members who had fallen ill. Other teachers might have even needed to cope with the death of family members, friends, or colleagues. During the months of lockdown, numerous teachers had to manage these challenges while being isolated from friends and family members. All of these factors may have contributed to remote teaching quality not always being optimal. Parents in a number of European countries stated that they were dissatisfied with the poor quality of homeschooling offer (Thorell et al., 2021). As a consequence, many teachers probably had to handle negative feedback from students and parents on top of their already complex workload. Thus, teachers have faced manifold challenges during the COVID-19 pandemic that may have exacerbated stress and even burnout.

The aim of the present review is to shift the spotlight from students to teachers and summarize the existing empirical findings on K−12 teachers' stress and burnout during the COVID-19 pandemic. The following questions guided our research:

1. To what extent did K−12 teachers' levels of stress and burnout increase during the COVID-19 pandemic?
2. Did K−12 teachers experience higher levels of stress and burnout than individuals employed in other occupational fields during the COVID-19 pandemic?
3. Which job and organizational characteristics were associated with higher levels of stress and burnout in K−12 teachers during the COVID-19 pandemic?
4. Which individual characteristics and activities were associated with higher levels of stress and burnout in K−12 teachers during the COVID-19 pandemic?

## Methods

### Inclusion criteria

Based on a preliminary literature search, we developed the following criteria for the inclusion of studies: studies (1) had to have measured teacher burnout or stress during the COVID-19 pandemic (2) in quantitative terms and (3) had to focus on K−12 teachers. They had to be published (4) in English (5) between 2020 (when the pandemic began) and 2021 (when we conducted the literature search). For inclusion in our review on research question 1—which focuses on the effects of the COVID-19 pandemic on teacher stress and burnout—the studies also had to have a longitudinal design with one measurement before and one measurement during the COVID-19 pandemic. For inclusion in our review based on research question 2, the studies had to report differences between K−12 teachers and individuals working in other professions. For inclusion in our review based on research questions 3 and 4, the studies had to report the association with another construct (that was not teacher stress or burnout). We did not include intervention studies without control groups, reviews, or non-empirical studies, e.g., opinion papers.

### Literature search and selection of studies

**Figure 1:**
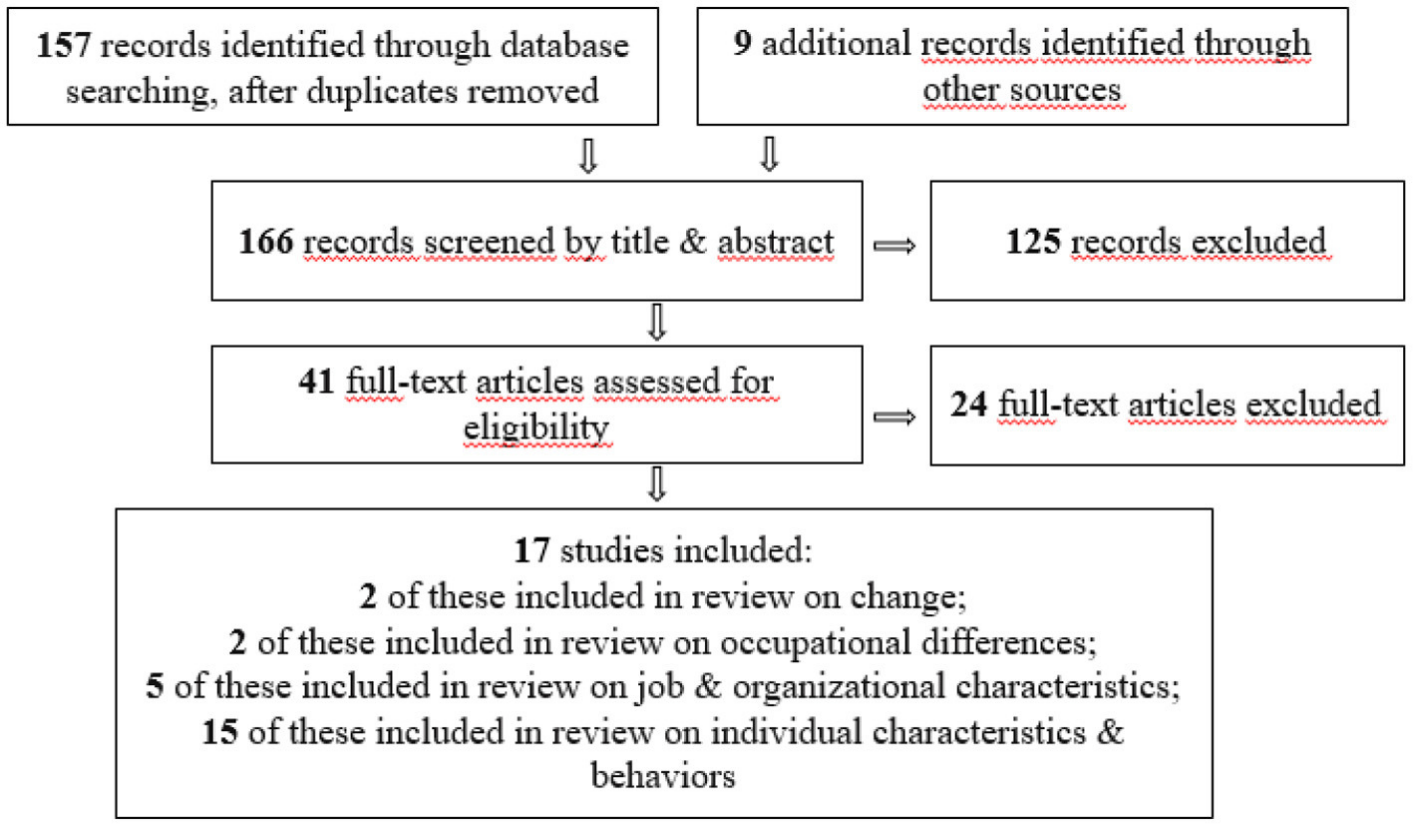
Literature search process with numbers of articles considered. When screening records by title and abstract, most of the records excluded did either not focus on K−12 teachers, did not apply a quantitative research design or were not written in English. Most of the full-text articles excluded had either not measured teacher burnout or stress or had not sampled K−12 teachers.

When searching for relevant studies, we used a multistep process (see Figure 1). In a first step, we developed a comprehensive search string, including words to describe the COVID-19 pandemic, words to describe stress or burnout, and the term “teachers.” We used the following search string: (Covid OR Corona OR “SARS-CoV-2” OR pandemic) AND teacher AND (stress OR distress OR burnout OR exhaustion OR disengagement OR depersonalization OR “reduced personal accomplishment” OR “reduced efficacy”). Using this syntax, we searched titles, abstracts, and keywords in the database Web of Science. The search was conducted in July 2021. There was no preselection of studies based on a rubric. To allow for the inclusion of studies that had not yet been accepted or had not undergone peer-review, we also searched the preprint archives EdArXiv, PsyArXiv, and SocArXiv, using the same search terms. This literature search yielded 157 studies.

The titles and abstracts of all 157 studies identified were thoroughly examined by the authors on the basis of the inclusion criteria. To test for interrater agreement, the first and second author each rated a subset of 20 studies. Inter-rater reliability was Cohen's *d* = 0.89. In case of disagreement, the authors discussed the studies in question until they reached consensus. During this first step, 41 studies were identified as being potentially eligible for our review. Nearly all of the studies that we excluded in this first step did either not focus on K−12 teachers, did not apply a quantitative research design or were not written in English. We subsequently read the full texts of all of the 41 eligible studies and decided upon inclusion based on our inclusion criteria.

To find additional relevant work, we conducted a backward reference search in the articles selected; that is to say, we examined whether other potentially relevant studies were cited in these articles. The selection process was repeated on the nine additional studies identified during this search. In total, 17 studies were selected for inclusion in our review during full-text screening. These studies were then coded by the first and second author and the coding was checked by a research assistant (see Table 1). Most of the studies that we excluded in this second step either had not measured teacher burnout or stress or had no K−12 teachers sample, which only became clear after reading the full-text.

**Table 1:** Description of non-intervention studies included in the review.

**Code**	**References**	**Country**	**Sample size**	Participants*	Time of data assessment**	Measure of Burnout/ Stress***	Measures of other relevant study constructs***	**Statistical method**	**Results**
A	Amri et al. (2020)	Morocco	*N*_teachers_ = 125	*M*_age_ = 38.6 (*SD* = 9.9); 57% female; only primary school teachers; professional seniority in years: *M* = 13.9 (*SD* = 8.9)	Apr and May 2020. Since Mar 2020: distance education in all educational establishments; during data collection period: all teachers were teaching remotely.	Arabic version of the MBI (Maslach and Jackson, 1981): EE, DP, sense of PA, 16 items out of org. 22 adapted to the context of Moroccan teachers; α ≥ 0.75	Requirements and resources related to distance education (developed by research team): workload, work-family conflicts, use and development of ICT (information and communication technologies) skills, social support; α ≥ 0.71; marital status: married, unmarried; age in years: 25–40, 41–59; professional seniority in years: <20, ≥ 20; gender	Chi-square tests; logistic regression	Chi-square test: factors significantly linked to burnout: [high] workload, [high] work-family conflicts, [low] use and development of ICT skills and [low] social support, age over 41 years and professional seniority over 20 years, but not gender and marital status. Logistic regression: significant risk factors for burnout are the [low] use and development of ICT skills, [high] work-family conflict, [low] social support and [high] workload, but not age or professional seniority.
B	Carreon et al. (2021)	Philippines	*N*_teachers_ = 1,069	Age in years: 56% ≤ 34, 25% 35–44, 20% ≥ 45; 80% female; years of teaching experience: 74% 0–10, 16% 11–20, 10% ≥ 21	Jan–Feb 2021	Online Teaching Burnout Tool (Panisoara et al., 2020) to measure burnout caused by remote teaching, items adapted to the context of remote teaching; α = 0.90	Fear of COVID-19 Scale (Ahorsu et al., 2020); α = 0.91; age; gender; teaching experience	Test of group differences in remote teaching burnout according to age, gender, and teaching experience (t test and ANOVA); bivariate correlation	Significant differences in remote teaching burnout for age between groups of ≤ 34 years (*M* = 2.95) and ≥ 45 years (*M* = 3.24) and between 15–44 years (*M* = 2.91) and ≥ 45 years (*M* = 3.24), as well as for teaching experience between groups of 0–10 years (*M* = 2.93) and ≥ 21 years (*M* = 3.31); no significant difference in remote teaching burnout according to gender. Significant correlation between fear of COVID-19 and remote teaching burnout (*r* = 0.44).
C	Collie (2021)	Australia	*N*_teachers_ = 325	*M*_age_ = 39 (*SD* = 12); 67% female, 31% male, 2% non-binary or other gender identity; 48% primary school teachers, 39% secondary school teachers, 13% both primary and secondary school teachers; years of teaching experience: *M* = 12 (*SD* = 11)	May 2020. During data collection period: various stages of restrictions for schooling in Australia's states and territories; 41% of teachers questioned were teaching fully remotely due to COVID-19, 21% were teaching half remotely due to COVID-19, 29% were teaching in-person in school, 2% were teaching remotely as usual and 6% were not teaching due to COVID-19.	MBI-EE (Maslach and Jackson, 1981), shortened; Stress Related to Change (Putwain and von der Embse, 2019), three items adapted to be relevant to general changes in teachers work; ω ≥ 0.78	Autonomy-Supportive Leadership (developed for this study) to measure job resource; Autonomy-Thwarting Leadership (developed for this study) to measure job demand; Workplace Buoyancy (Martin and Marsh, 2008) to measure personal resource; Somatic Symptom Scale (Gierk et al., 2014) to assess somatic burden; Big Five Personality Dimensions (Gosling et al., 2003): openness, constraint, extraversion, neuroticism; ω ≥ 0.79; gender; teaching experience; school location: rural/remote, urban/suburban; working situation: teaching fully remotely, half remotely, reduced work due to COVID-19	Bivariate correlations; structural equation modeling	EE significantly correlated with autonomy-supportive leadership and workplace buoyancy (-0.30 ≥*r* ≥−0.34), with somatic burden (*r* = 0.35), autonomy-thwarting leadership, neuroticism, and stress (0.50 ≤ *r* ≤ 0.59); all other correlations with EE were non-significant. Stress significantly correlated with autonomy-thwarting leadership, teaching half remotely, urban/suburban school location (0.15 ≤ *r* ≤ 0.16), openness, constraint, neuroticism and somatic burden (0.31 ≤ *r* ≤ 0.48), all other correlations with stress were non-significant. Direct significant associations of workplace buoyancy (β = −0.29), neuroticism (β = 0.29), autonomy-thwarting leadership and constraint (0.34 ≤ β ≤ 0.46) with EE; significant indirect association of autonomy-supportive leadership with EE, mediated by workplace buoyancy (β = −0.12); all other associations with EE were non-significant. Direct significant associations of gender [female] (β = −0.13), teaching half remotely (β = 0.10), workplace buoyancy (β = −0.37), openness, neuroticism (0.31 ≤ β ≤ 0.33), and constraint (β = 0.57) with stress; significant indirect association of autonomy-supportive leadership with stress, mediated by workplace buoyancy (β = −0.16); all other associations with stress were non-significant.
D	Liu et al. (2021)	China	*N*_teachers_ = 449	*M*_age_ = 36.7 (*SD* = 2.3); 74% female; only high school teachers	Nov 2020–Jan 2021	MBI (Maslach and Jackson, 1981), shortened: EE, DP, low PA, response scale adapted (1 = very inconsistent, 5 = very consistent); α ≥ 0.71	Connor-Davidson Resilience Scale (CD-RISC; Connor and Davidson, 2003): confidence, optimism, strength; α = 0.96, α for subscales =0.66–0.93; Turnover Intention Scale (Price, 2001); α ≥ 0.84	Bivariate correlations; pairwise regressions; structural equation model	Job burnout (-0.38 ≥*r* ≥−0.47), EE (-0.36 ≥*r* ≥−0.43), DP (-0.33 ≥*r* ≥−0.47) and low PA (-0.29 ≥*r* ≥−0.36) are significantly correlated with all dimensions of resilience; job burnout (*r* = 0.49), EE (*r* = 0.44), DP (*r* = 0.48) and low PA (*r* = 0.33) are significantly correlated with turnover intention. Dimensions of resilience significantly predict EE (-0.24 ≥β ≥−0.38), DP (-0.37 ≥β ≥−0.45) and low PA (-0.40 ≥β ≥−0.50); EE (β = 0.43), DP (β = 0.17) and low PA (β = 0.11) significantly predict turnover intention. Structural equation model: significant direct effects of resilience on job burnout (λ = −0.54) and of job burnout on turnover intention (λ = 0.52).
E	Ma et al. (2021)	China	*N*_teachers_ = 351	67% female; 42% senior high school teachers, 44% junior high school teachers, 12% primary school teachers; years of teaching experience: 29% 15–20, 14% 10–15, 17% 5–10, 41% 0–5; 55% advanced school teachers	Aug 2020 during summer semester break [participants retrospectively reported their online TSE (Teacher Self-Efficacy) at the beginning (T_retro_1) and end (T_retro_2) of online teaching during school lockdown].	Job Burnout Inventory for Secondary Teachers (Wang et al., 2003), shortened: passion burnout, energy burnout, professional self-effectiveness burnout; α ≥ 0.75	Michigan Nurse Educators Sense of Efficacy for Online Teaching Survey (Robinia, 2008): TSE for online instruction, TSE for technology application in online teaching; Adaptability Scale (Martin et al., 2012); α ≥ 0.78	Bivariate correlations of burnout with TSE and adaptability at T_retro_1 and with TSE at T_retro_2	Significant correlations between passion burnout and TSE for online instruction (T_retro_1 *r* = −0.13; T_retro_2 *r* = −0.14), TSE for technology application (T_retro_1 *r* = −0.14; T_retro_2 *r* = −0.18) and adaptability (*r* = −0.19). Significant correlations between reduced effectiveness and TSE for online instruction (T_retro_1 *r* = −0.27; T_retro_2 *r* = −0.31), TSE for technology application (T_retro_1 *r* = −0.23; T_retro_2 *r* = −0.27), and adaptability (*r* = −0.39). No significant correlations between energy burnout and the two TSE-domains at any time (*r* ≤ 0.10). Significant correlation between energy burnout and adaptability (*r* = 0.13).
					In China, majority of schools commenced online teaching in mid Feb 2020; gradual reopening of schools with de-escalation of COVID-19 situation; until mid Aug 2020, ~ 75% of students returned to schools.				
F	Mari et al. (2021)	Italy	*N*_participants_ = 628	*M*_age_ = 42.3 (*SD* = 10.5); 78% female; 26% practitioners (lawyer, psychologist, accountant etc.), 20% managers, 29% executive employees, 25% teachers	Apr 2020. Italian government imposed lockdown restrictions including school closures in Mar 2020; during data collection: all participants worked virtually.	Italian translation of the PSS (Cohen et al., 1983; Fossati, 2010): perceived self-efficacy, perceived helplessness; α ≥ 0.75	Groups: practitioners, managers, executive employees, teachers	One-way analysis of variance (ANOVA) comparing groups in terms of perceived stress; *post-hoc* analysis using Bonferroni correction	No significant differences in total PSS between groups; no significant differences in perceived self-efficacy between groups; significant differences in perceived helplessness: teachers have a significant higher score (*M* = 11.07, *SD* = 3.9) than managers (*M* = 9.79, *SD* = 3.8), apart from that no significant differences in perceived helplessness.
G	Oducado et al. (2021)	Philippines	*N*_teachers_ = 105	*M*_age_ = 33.9 (*SD* = 8.8); 85% female	Aug 2020	COVID-19 PSS (COVID-PSS-10; Pedrozo-Pupo et al., 2020); α = 0.83	Single item on self-rated health (Haddock et al., 2006); single item on perceived risk of getting infected with COVID-19; gender; age	Test of group differences in perceived stress according to gender (Mann-Whitney *U*-test and Kruskal-Wallis test); bivariate correlations	Significant differences in perceived stress according to gender (male *Mean Rank* = 38.41; female *Mean Rank* = 55.62). Significant correlations of perceived stress with self-rated health (*rho* = −0.27) and perceived risk of getting infected (*rho* = 0.42), but not with age.
H	Ozamiz-Etxebarria et al. (2021)	Spain (Basque Autonomous Community)	*N*_teachers_ = 1,633	*M*_age_ = 42.6 (*SD* = 10); 80% female; 19% pre-school teachers, 33% primary school teachers, 30% secondary school teachers, 6% bachelor studies teachers, 6% teachers for vocational training, 8% university studies teachers	Sep 2020. In Spain, schools and universities were closed in Mar 2020; in Sep 2020, most schools and universities reopened.	Stress subscale of Spanish version of Depression Anxiety and Stress Scale-21 (DASS-21; Ruiz et al., 2017); α ≥ 0.75	Teaching sector: pre-school, primary school, secondary school, bachelor studies, vocational training, university studies	Test of group differences in stress according to teaching sector (ANOVA)	No significant group differences in stress for teachers in different teaching sectors.
I	Panisoara et al. (2020)	Romania	*N*_teachers_ = 980	Age in years: 20–68; 97% female	Apr 2020 [n_1_ = 462]; Apr–May 2020 [n_2_ = 518]. In Romania, online teaching was mandatory in all schools from 16 Mar 2020.	Oldenburg Burnout Inventory (OBI; Demerouti et al., 2003); Person–Technology-Enhanced Learning Misfit Scale (P–TEL; Wang et al., 2020) to measure technostress; both instruments were adapted to the context of online teaching and translated into Romanian; α ≥ 0.83 [Based on the results of exploratory factor analysis, burnout and technostress are combined into a single construct for further analyses]	Work Tasks Motivation Scale for Teachers (WTMST; Fernet et al., 2008) to measure intrinsic and extrinsic motivation, items were adapted; Continuance Intention Scale (CI; Akbulut, 2009; Wu and Chen, 2017; Wang et al., 2020) to measure continuance intention to use online instruction; Technological Pedagogical Knowledge (TPK) Self-Efficacy (Chuang et al., 2018; Iliescu et al., 2019), items were modified; α ≥ 0.88, except for extrinsic motivation: α = 0.68	Bivariate correlations (only for n_2_); path analysis	Significant correlations of burnout and technostress with TPK self-efficacy, intrinsic motivation and CI (-0.35 ≥*r* ≥−0.55); significant correlation of burnout and technostress with extrinsic motivation (*r* = 0.55). Path analysis: significant, but weak direct effect of burnout and technostress on CI (β = 0.06). Burnout and technostress were significantly affected by intrinsic motivation (β = −0.36) and extrinsic motivation (β = 0.48), but not by TPK self-efficacy.
J	Pressley (2021)	United States	*N*_teachers_ = 359	Years of teaching experience: *M* = 13.3 (*SD* = 9.1)	Oct 2020. During data collection period: teachers faced different teaching conditions, including socially distanced classrooms, hybrid teaching, or 100% virtual instruction.	Teacher Burnout Scale (Seidman and Zager, 1986): subscales administrative support, stress; α ≥ 0.80	COVID Anxiety Scale (CAS; Lee, 2020); α = 0.90; 7 one-item anxiety questions (Davey et al., 2007) focused on teaching during fall 2020: anxiety the first week, anxiety using district technology, anxiety providing virtual instruction, current anxiety, anxiety communicating with parents, anxiety communicating with administrators; ethnicity; location: rural, urban, suburban; type of instruction: face-to-face, hybrid, virtual	Bivariate correlations; hierarchical multiple regression model	Stress is significantly correlated with anxiety the first week, anxiety using district technology, anxiety providing virtual instruction (0.16 ≤ *r* ≤ 0.25), current anxiety, anxiety communicating with parents, anxiety communicating with administrators and CAS (0.34 ≤ *r* ≤ 0.50), but not with teaching experience. Administrative support is significantly correlated with current anxiety, anxiety communicating with parents, CAS (0.14 ≤ *r* ≤ 0.20) and anxiety communicating with administrators (*r* = 0.54), but not with teaching experience and all other teaching anxiety items. CAS (β = 0.31), current anxiety (β = 0.35), anxiety communicating with parents (β = 0.17), administrative support (*β =* 0.21), and teaching experience (β = 0.10) are significant predictors for stress, but not ethnicity, location, type of instruction and all other teaching anxiety items.
K	Rabaglietti et al. (2021)	Italy (IT) and other European countries (OEC)	*N*_teachers_ = 366	*M*_age_ = 45.3 (*SD* = 10.4); 86% female; 55% teachers from IT, 45% teachers from OEC; years of teaching experience: teachers from IT: *M* = 14.7 (*SD* = 11.5), teachers from OEC: *M* = 17.6 (*SD* = 10.9)	Spring 2020. During data collection period: in IT, all teachers had already been teaching remotely for several weeks; in OEC, distance learning had just started, in some cases limited to certain school grades or geographical areas.	PSS (Cohen et al., 1983), wording of the introductory sentence adapted to “since the period of social distancing;” α = 0.86	Difficulties in Organizing Distance Learning (DDL; created *ad hoc*); General Self-Efficacy Scale (GSE; Schwarzer and Jerusalem, 1995; Sibilia et al., 1995); α ≥ 0.74; sample groups: IT, OEC	Bivariate correlations; path analysis / mediation analyses; test of differences between both samples	Significant correlations between PSS and GSE (total sample *r* = −0.35; IT *r* = −0.49; OEC *r* = −0.50) and between PSS and DDL (total sample *r* = 0.45; IT *r* = 0.49; OEC *r* = 0.44). Significant direct effect of DDL on PSS (*B* = 1.14, *SE* = 0.15) and of GSE on PSS (*B* = −0.56, *SE* = 0.07). Significant indirect effect of DDL on PSS, mediated by GSE (*B* = 0.42, *SE* = 0.09). Significant difference between both samples: effect of GSE on PSS was stronger for IT teacher (IT *B* = −0.88, *SE* = 0.10; OEC *B* = −0.41, *SE* = 0.08). Neither the direct effect nor the indirect effect of DDL on PSS were significantly different between the two samples.
L	Sokal et al. (2020a)	Canada	*N*_teachers_ = 1,626	Age in years: 3% <25, 14% 26–30, 32% 31–40, 32% 41–50, 19% > 50; 84% female; years of teaching experience: 3% <1, 15% 2–5, 19% 6–10, 21% 11–15, 42% > 15	Apr 2020 [*N* = 1,278]; Jun 2020 [*N* = 348]. During data collection period: all teachers were teaching remotely.	MBI for Educators (Maslach and Jackson, 1981; Maslach et al., 1996): exhaustion, cynicism, PA, response scale adapted (1 = a few times since beginning teaching at home); α not reported	Teacher Sense of Self-Efficacy Scale (Tschannen-Moran and Woolfolk Hoy, 2001): efficacy with strategies, with behavior management, with engagement; Attitudes Toward Technology (Edison and Geissler, 2003): subscale and statement to reflect current change to technology-based teaching; Teacher Attitudes Toward Change (TATC) Scale (Kin and Kareem, 2017): cognitive, affective, and behavioral responses to change; resilience (Eddy et al., 2019): subtraction of a single item on perceived stress from a single item on perceived coping with stress; α not reported	Bivariate correlations	Significant correlations between exhaustion and resilience (*r* = −0.80), all dimensions of self-efficacy (-0.12 ≥*r* ≥−0.20), all dimensions of TATC (-0.10 ≥*r* ≥−0.29) and attitudes toward technology (*r* = −0.25). Significant correlations between cynicism and resilience (*r* = −0.30), all dimensions of self-efficacy (-0.20 ≥*r* ≥−0.32) and all dimensions of TATC (-0.10 ≥*r* ≥−0.22), but not with attitudes toward technology. Significant correlations between PA and resilience (*r* = 0.24), all dimensions of self-efficacy (0.38 ≤ *r* ≤ 0.61), all dimensions of TATC (0.20 ≤ *r* ≤ 0.33) and attitudes toward technology (*r* = 0.17).
M	Sokal et al. (2020b)	Canada	*N*_teachers_ = 1,278	Age in years: 3% <25, 15% 26–30, 32% 31–40, 33% 41–50, 18% > 50; 84% female; years of teaching experience: 3% <1, 17% 2–5, 19% 6–10, 20% 11–15, 41% > 15	Apr–May 2020. During data collection period: all teachers were teaching remotely.	MBI for Educators (Maslach and Jackson, 1981; Maslach et al., 1996): exhaustion, cynicism, PA, response scale adapted (1 = a few times since beginning teaching at home); α not reported	List of anticipated job demands: parental expectations, work/life balance, time management, lack of resources, technology issues; list of anticipated job resources: support from administrators, parents, peers, partner/family or friends, instruction on new methods or technology, exercise, sleep, healthy eating, meditation, prayer, counseling/therapy, journaling, mindfulness; α not reported	Bivariate correlations	Correlations between burnout and job demands: significant, except cynicism with technology issues and PA with time management. Moderate correlations between exhaustion and time management, technology issues and work/life balance (0.31 ≤ *r* ≤ 0.45), small correlations between exhaustion and parental expectations and lack of resources (0.20 ≤ *r* ≤ 0.23), between cynicism and time management, work/life balance and lack of resources (0.10 ≤ *r* ≤ 0.13), as well as between PA and lack of resources (*r* = −0.24). All other correlations of burnout with job demands |*r|* ≤ 0.07. Correlations between burnout and job resources: small significant correlations between exhaustion and support from partner/family, support from friends, instruction on technology, and counseling/therapy (0.10 ≤ *r* ≤ 0.15), between cynicism and support from administrators, and support from parents (-0.10 ≥*r* ≥−0.11), as well as between PA and support from administrators, support from parents, support from partner/family, instruction on technology, exercise, healthy eating, and mindfulness (0.10 ≤ *r* ≤ 0.18).
									Very small, but significant correlations between exhaustion and support from administrators (*r* = −0.07), support from peers, instruction on new methods, meditation, and journaling (0.06 ≤ *r* ≤ 0.09), between cynicism and exercise, healthy eating, mindfulness (-0.06 ≥*r* ≥−0.07), and counseling/therapy (*r* = 0.09) as well as between PA and support from peers, instruction on new methods, meditation, prayer, and journaling (0.06 ≤ *r* ≤ 0.09). All other correlations of burnout with job resources were non-significant.
N	Weißenfels et al. (2021)	Germany	*N*_teachers_ = 92. *N*_schools_ = 23	*M*_age_ = 40.2 (*SD* = 9.6); 82% female; 58% primary school teachers, 42% secondary school teachers	T1: Oct–Dec 2019; T2: started mid May 2020. In Germany, schools were gradually reopened after a period of online teaching in May 2020.	MBI (Maslach et al., 1986): EE, DP, lack of accomplishment; α ≥ 0.73, except for DP at T2: α = 0.68	Scale for Teacher Self-Efficacy (TSE; Pfitzner-Eden et al., 2014): classroom management, instructional strategies, student engagement; Teacher Self-Efficacy for Using Digital Media; Attitudes Toward E-Learning (Mishra and Panda, 2007); α ≥ 0.77, except for TSE instructional strategies: α ≥ 0.65	Latent change regression models	Significant means of difference scores for lack of accomplishment (*M* = 0.71, *SE* = 0.15) and DP (*M* = 0.74, *SE* = 0.23); no significant mean difference score for EE). Increase in MBI subscales is related to less increase in TSE subscales (-0.13 ≥*r* ≥−0.41); significant correlation of TSE for using digital media with the change in lack of accomplishment (*r* = −0.21), all other correlations between TSE for using digital media and attitudes toward e-learning respectively with changes in dimensions of burnout were non-significant.
O	Zhou and Yao (2020)	China	*N*_teachers_ = 751	*M*_age_ = 40 (*SD* = 8.4); 34% female; secondary and primary school teachers	Started Mar 2020. During data collection period: all teachers were teaching remotely.	Diagnostic and Statistical Manual of Mental Disorders (DSM)-5 Acute Stress Disorder Diagnostic Criteria B (American Psychiatric Association, 2013), 14 items; α = 0.86	Revised Received Social Support Questionnaire (Zhen et al., 2018); Basic Psychological Needs Scale (Sheldon and Niemiec, 2006): needs for autonomy, competence, relatedness; Sense of Control Subscale in Feelings of Safety Scale (An et al., 2004); α ≥ 0.80	Bivariate correlations; path model	Significant correlations of stress with all types of psychological needs (-0.22 ≥*r* ≥−0.30) and sense of control (*r* = −0.41), but not with social support. Significant direct associations of stress with sense of control (β = −0.33), and the needs for autonomy (β = −0.15) and relatedness (β = −0.11), but not with social support or needs for competence. Significant indirect relation of social support with stress *via* the needs for autonomy or relatedness (-0.03 ≥β ≥−0.04), and *via* the paths from all types of psychological needs to sense of control (-0.01 ≥β ≥−0.02).

*Mean age and years of teaching, gender ratio, type of school, possibly focus on specific subject.

**Month, year, possibly information about school closures in specific country and region and information about working conditions of teachers.

***Name of scales: dimensions; reliability of measures.

MBI, Maslach Burnout Inventory; PSS, Perceived Stress Scale; EE, emotional exhaustion; DP, depersonalization; PA, personal accomplishment.

Percentages can add up to more than 100% due to rounding. Significant: p < 0.05. All studies used online surveys to collect data. Studies A (Amri et al., 2020) and J (Pressley, 2021) were brief manuscripts of five pages or less.

The quality of the studies included in the review was independently rated by two of the authors based on eight quality indicators for non-intervention studies and 14 quality indicators for intervention studies (adapted from Hwang et al., 2017; see Table 2). In case of disagreement, studies were discussed until consensus was reached. We did not exclude any studies based on the quality ratings. Instead, these ratings serve as indicators of the overall quality of the research (see Tables 3, 4).

**Table 2:** Description of intervention studies included in the review.

**Code**	**References**	**Country**	**Sample size**	Participants*	Time of data assessment**	**Intervention (name, duration and content)**	Measure of burnout or stress***	Measures of other relevant study constructs***	**Statistical method**	**Results**
P	Pozo-Rico et al. (2020)	Spain	*N*_teachers_ = 141 (intervention group = 70, control group = 71)	*M*_age_ = 38.4 (*SD* = 7.0); 55% female; primary school teachers; years of teaching experience: *M* = 13.1 (*SD* = 6.8)	T1: pretest two weeks before intervention; T2: posttest two weeks after intervention. During data collection period: Spanish population was confined.	14-week teacher training program; teacher training program intended to improve stress management, prevent burnout in the teaching profession, improve competency and use of ICT to support teaching and learning and introduce pedagogical principles based on emotional intelligence into the classroom	Spanish version of PSS (Cohen et al., 1983); Spanish version of MBI (Maslach et al., 1986): EE, DP, PA; α ≥ 0.74	None	Multivariate analysis of variance; univariate analysis of variance of repeated measures	Significant interaction between evaluation time (pre-test and post-test) and intervention for all variables: Compared to the changes in the control group, intervention group showed a significant decrease in PSS (partial *η^2^* = 0.66), EE (partial *η^2^* = 0.63), DP (partial *η^2^* = 0.76) and an increased sense of PA (partial *η^2^* = 0.46).
Q	Zadok-Gurman et al. (2021)	Israel	*N*_teachers_ = 67 (intervention group = 35, control group = 32) dropouts = 7 (intervention group = 3, control group = 4)	*M*_age_ = 45; 87% female; years of teaching experience: *M* = 17	T1: baseline; T2: after intervention; intervention: Nov 2019–May 2020. Mar 2020: start of first lockdown due to COVID-19 pandemic.	Inquiry-Based Stress Reduction (IBSR) intervention: 10 biweekly group meetings (2.5 h each) and biweekly individual sessions with a facilitator (1 h each) for 20 weeks; all sessions were standardized according to a training manual; intervention program was moved to online format as of Mar 2020; step 1: participants identify stressful thoughts and write them down;				
						step 2: participants investigate their stressful thoughts using guided questions → enables them to question their automatic thoughts and examine their emotional and physical responses during stress-evoking situations, goal is realization, not rationalization; step 3: participants identify possible evidence for the opposite of the thought.	MBI (Maslach et al., 1996), shortened: EE, PA; PSS (Cohen, 1986); α ≥ 0.80	None	Mixed model analysis: pre-post x group.	Effects of IBSR intervention between the study groups: significant difference in EE (Cohen's *d* = 0.75): significantly less increase in EE in intervention group (T1 *M* = 12.7, *SD* = 5.9; T2 *M* = 18.3, *SD* = 5.4) than in control group (T1 *M* = 9.7, *SD* = 4.9; T2 *M* = 18.6, *SD* = 4.5); no significant differences in the change of PA and PSS between intervention group and control group.

*Mean age and years of teaching, gender ratio, type of school, possibly focus on specific subject.

**Month, year, possibly information about school closures in specific country and region and information about working conditions of teachers.

***Name of scales: dimensions; reliability of measures.

MBI, Maslach Burnout Inventory; PSS, Perceived Stress Scale; EE, emotional exhaustion; DP, depersonalization; PA, personal accomplishment.

Significant: p < 0.05. All studies used online surveys to collect data.

**Table 3:** Quality ratings of non-intervention studies included in the review.

**References**	**Aims and objectives**	**Sufficient sample information**	**Reliability of burnout/ stress measures**	**Validity of burnout/ stress measures**	**Reliability of other relevant study constructs**	**Ethical consideration**	**Alignment of research question(s) and data analysis**	**Clear structure of manuscript**
Amri et al. (2020)	3	3	3	1	3	1	2	2
Carreon et al. (2021)	3	2	3	1	3	1	2	2
Collie (2021)	3	3	3	3	3	3	3	3
Liu et al. (2021)	3	2	3	2	3	3	2	2
Ma et al. (2021)	3	3	3	3	3	2	1	3
Mari et al. (2021)	2	2	3	2	–	3	1	3
Oducado et al. (2021)	2	2	3	3	–	1	1	3
Ozamiz-Etxebarria et al. (2021)	2	3	3	3	–	3	3	3
Panisoara et al. (2020)	3	2	3	1	3	3	3	2
Pressley (2021)	2	2	3	2	3	1	1	3
Rabaglietti et al. (2021)	3	3	3	3	3	3	2	3
Sokal et al. (2020a)	3	3	1	3	1	3	1	2
Sokal et al. (2020b)	3	3	1	3	1	3	2	3
Weißenfels et al. (2021)	3	3	3	3	3	2	3	3
Zhou and Yao (2020)	3	2	3	3	3	3	3	3

Aims and objectives: 3 = clear and comprehensible research question(s) or hypotheses; 2 = limitations in clarity and comprehensibility of research question(s) or hypotheses; 1 = no clear and comprehensible research question(s) or hypotheses.

Sufficient sample information, i.e., sample size, country, age, gender, school type, professional experience, teaching remotely: 3 = all information available or one of these aspects missing; 2 = two to three of these aspects missing; 1 = four or more of these aspects missing.

Reliability of burnout/stress measures: 3 = reliability reported and acceptable (α ≥ 0.65); 2 = reliability reported but not acceptable (α <0.65); 1 = reliability not reported.

Validity of burnout/stress measures: 3 = completely valid; 2 = minor limitations in validity; 1 = major limitations in validity.

Reliability of other relevant study constructs: 3 = reliability reported and acceptable (α ≥ 0.65); 2 = reliability reported but not acceptable (α <0.65); 1 = reliability not reported; – = no other scales used.

Ethical consideration: 3 = approval of ethics commission; 2 = no approval of ethics commission required according to the authors; 1 = no approval of ethics commission reported.

Alignment of research question(s) and data analysis: 3 = robust analyses and data that answer research question(s) or hypotheses; 2 = minor limitations in data and/or analyses; 1 = data and/or analyses exhibit major limitations and do not answer research question(s) or hypotheses.

Clear structure of manuscript: 3 = clear structure according to APA-standards; 2 = minor limitations in structure according to APA-standards; 1 = major limitations in structure according to APA-standards.

**Table 4:** Quality ratings of intervention studies included in the review.

**References**	**Aims and objectives**	**Sufficient sample information**	**Random assignment of participants**	**Similarity between groups**	**Information about intervention**	**Measurement at appropriate times**	**Information about comparison conditions**	**Fidelity of intervention**	**Effect size reported**	**Reliability of burnout/stress measures**	**Validity of burnout/stress measures**	**Ethical consideration**	**Alignment of research question(s) and data analysis**	**Clear structure of manuscript**
Pozo-Rico et al. (2020)	3	3	3	3	3	2	3	1	3	3	3	3	3	3
Zadok-Gurman et al. (2021)	3	2	2	1	3	1	2	3	3	3	3	3	2	3

Aims and objectives: 3 = clear and comprehensible research question(s) or hypotheses; 2 = limitations in clarity and comprehensibility of research question(s) or hypotheses; 1 = no clear and comprehensible research question(s) or hypotheses.

Sufficient sample information, i.e., sample size, country, age, gender, school type, professional experience, teaching remotely: 3 = all information available or one of these aspects missing; 2 = two to three of these aspects missing; 1 = four or more of these aspects missing.

Random assignment of participants: 3 = participants were randomly assigned to conditions or control group was matched to treatment group; 2 = participants were non-randomly assigned to conditions and no matching prior to treatment; 1 = assignment to condition not described or vague.

Similarity between intervention and control group at the start of the intervention: 3 = similarity of groups given or differences controlled for in analyses; 2 = limited similarity of groups; 1 = significant differences between groups not controlled for in analyses or no information on similarity of groups.

Information about intervention (e.g., name, duration, content): 3 = sufficient information; 2 = little information; 1 = no information.

Measurement of intervention effect at appropriate times: 3 = pre-, post- and follow-up-test conducted; 2 = pre- and post-test conducted, 1 = time of post-test not exactly reported.

Information about comparison conditions, 3 = sufficient information or no treatment; 2 = little information; 1 = no information.

Fidelity of intervention: 3 = good fidelity of implementation reported; 2 = limited fidelity of implementation reported; 1 = poor fidelity or no information on fidelity reported.

Effect size reported: 3 = reported; 2 = not reported, but can be calculated from other reported measures; 1 = not reported and no other measures allowing calculation.

Reliability of burnout / stress measures: 3 = reliability reported and acceptable (α ≥ 0.65); 2 = reliability reported but not acceptable (α <0.65); 1 = reliability not reported.

Validity of burnout/stress measures: 3 = completely valid; 2 = minor limitations in validity; 1 = major limitations in validity.

Ethical consideration: 3 = approval of ethics commission; 2 = no approval of ethics commission required according to the authors; 1 = no approval of ethics commission reported.

Alignment of research question(s) and data analysis: 3 = robust analyses and data that answer research question(s) or hypotheses; 2 = minor limitations in data and/or analyses; 1 = data and/or analyses exhibit major limitations and do not answer research question(s) or hypotheses.

Clear structure of manuscript: 3 = clear structure according to APA-standards; 2 = minor limitations in structure according to APA-standards; 1 = major limitations in structure according to APA-standards.

## Results

To give an overview of the included studies, we first present (3.1) the measures of K−12 teacher stress and burnout (3.2), the research designs, and (3.3) the teacher samples used in the studies. We then report (3.4) the study findings on changes in K−12 teachers' levels of stress and burnout during the COVID-19 pandemic and (3.5) differences in stress and burnout between K−12 teachers and individuals employed in other occupational fields. Finally, we outline findings on the relevance of (3.6.1) job and organizational characteristics and (3.6.2) individual characteristics. In Table 1, we present details about the time frame and the country, in which the study was conducted.

### Measures of K−12 teacher stress and burnout

Of the 17 studies included in our review, three studies assessed both teacher stress and burnout, nine studies just assessed teacher burnout, and five studies focused only on teacher stress. Of the 12 studies examining burnout, eight relied on Maslach's operationalization of burnout, applying the original Maslach Burnout Inventory (MBI; k = 6; Maslach et al., 1986) or the MBI-Educator Survey (k = 2; Maslach et al., 1996). Two studies (Panisoara et al., 2020; Carreon et al., 2021) selected and adapted items of the Oldenburg Burnout Inventory (Demerouti et al., 2003; both subscales: exhaustion and disengagement) to measure burnout in regard to distance learning. One study (Ma et al., 2021) administered an adapted version of the Job Burnout Inventory (Wang et al., 2003), a Chinese burnout inventory measuring the subscales passion burnout, energy burnout, and professional self-effectiveness burnout. Pressley (2021) used two teacher burnout subscales—assessing administration support and stress—of the Teacher Burnout Scale by Seidman and Zager (1986).

Of the eight studies measuring stress, four used the Perceived Stress Scale (PSS; Cohen et al., 1983) and one used an adapted version of this scale (COVID-PSS-10; Pedrozo-Pupo et al., 2020). One study (Ozamiz-Etxebarria et al., 2021) applied the Depression Anxiety and Stress Scale-21 (DASS-21; Ruiz et al., 2017), one study (Zhou and Yao, 2020) assessed diagnostic criteria of an acute stress disorder, based on in the Diagnostic and Statistical Manual of Mental Disorders (DSM-5; American Psychiatric Association, 2013), and one study (Collie, 2021) measured stress related to change (Putwain and von der Embse, 2019).

### Research designs

The majority of the studies (k = 14) included in our review applied a cross-sectional and non-experimental study design, surveying teachers once during the pandemic. One study (Weißenfels et al., 2021) surveyed teachers twice during the pandemic. Two studies (Pozo-Rico et al., 2020; Zadok-Gurman et al., 2021) applied intervention designs, in which changes in teachers' stress and burnout in an intervention group were compared to a control group.

### Teacher samples

A total of *N* = 9,874 teachers participated in the 17 studies included in the review. The number of participants per study ranged from 67 to 1,633. Participants were recruited in 20 countries all across the world, including the United States, Canada, Australia, China, Morocco, the Philippines, and a range of European countries. We present the teacher samples broken down by country in Table 5. Teachers' average age ranged from 33.9 to 45.3 years, 77.8% were female (ranging from 34.2% to 96.8%; k = 15; *N* = 9,358). Overall, 33.9% were primary school teachers (k = 7; *N* = 3,116). Five studies reported they recruited teachers from both primary and secondary schools (Zhou and Yao, 2020; Collie, 2021; Ma et al., 2021; Ozamiz-Etxebarria et al., 2021; Weißenfels et al., 2021). Two studies (Mari et al., 2021; Ozamiz-Etxebarria et al., 2021) compared K−12-teacher samples to samples of preschool teachers, university teachers, managers, and executive employees.

**Table 5:** K−12 teachers samples broken down by country.

**Country**	***N*_K–12_ teachers**	**%**
Australia	325	3.3
Austria	15	0.2
Canada	2,904	29.4
China	1,551	15.7
France	13	0.1
Germany	109	1.1
Hungary	15	0.2
Ireland	18	0.2
Italy	357	3.6
Israel	67	0.7
Latvia	10	0.1
Liechtenstein	8	0.1
Lithuania	22	0.2
Morocco	125	1.3
Netherlands	8	0.1
Philippines	1,174	11.9
Portugal	17	0.2
Romania	980	9.9
Spain	1,797	18.2
United States	359	3.6
Australia	325	3.3
Total	9,874	100.0

Samples used in more than one publication were taken into account only once. Study participants who were not K−12 Teachers (e.g., managers) have been excluded.

### Study findings on the changes in teacher stress and burnout during the pandemic

Two studies reported changes in teachers' stress and burnout levels during the pandemic compared to burnout levels prior to the pandemic (Weißenfels et al., 2021; control group in Zadok-Gurman et al., 2021). One study showed an increase in lack of accomplishment and depersonalization, but no change in emotional exhaustion (Weißenfels et al., 2021; T2 survey in May 2020; MBI; latent change regression), while the other study found an increase in emotional exhaustion (Cohen's *d* = 1.88),^1^ but no change in personal accomplishment or stress (control group in Zadok-Gurman et al., 2021; T2 survey in May 2020; MBI).

### Study findings on the differences in stress and burnout between K−12 teachers and individuals employed in other occupational fields

Two studies examined differences between K−12 teachers' stress levels and stress levels in other professions. Ozamiz-Etxebarria et al. (2021) found no differences in stress levels experienced by K−12 teachers in comparison to preschool teachers' and university teachers' stress levels (assessed in September 2020; Spain). Mari et al. (2021) found no differences in the PSS-subscale perceived self-efficacy between teachers, managers, executive employees, and other practitioners (i.e., lawyers, psychologists, accountants; assessed in April 2020; Italy). On the PSS-subscale helplessness, teachers reported higher scores than managers did (Cohen's *d* = 0.33),^2^ while there were no differences between teachers and the other professions.

### Study findings on the links between teacher stress and burnout and job, organizational, and individual characteristics

When presenting the study findings in the following, we will only include findings pertaining to teacher stress and burnout, although some studies reported additional results.

#### Job characteristics and organizational characteristics

##### Leadership

One study examined the role of leadership (Collie, 2021) and showed that autonomy-thwarting leadership was positively associated with emotional exhaustion (standardized beta = 0.46), but not with stress related to change. Autonomy-supportive leadership was not directly associated with emotional exhaustion or stress, but indirectly affected both stress and emotional exhaustion positively *via* workplace buoyancy.

##### Workload and amount of remote teaching

Two studies examined associations between teacher burnout and reduced work (hours per week) or self-reported workload (Amri et al., 2020; Collie, 2021), as well as the amount of remote teaching (Collie, 2021). Teacher burnout was associated with workload in one of the two studies. Having to teach a mix of in-person and online instruction was associated with higher stress, but not with emotional exhaustion (Collie, 2021).

##### Job demands and resources

Two studies examined the associations between teacher stress and burnout and job demands (Rabaglietti et al., 2021) and resources (Sokal et al., 2020b). Higher emotional exhaustion was associated with higher parental expectations, a lack of resources, technology demands, time-management issues, difficulties in balancing home and teaching, and more resources on instruction and on new methods and technology (Sokal et al., 2020b). These job demands and resources were also positively associated with accomplishment and cynicism, although associations were smaller (Sokal et al., 2020b). Similar demands were associated with stress (Rabaglietti et al., 2021).

##### School location

Two studies investigated the role of the school location (rural or remote vs. urban or suburban, Collie, 2021; rural vs. suburban vs. urban, Pressley, 2021) and found no association with the Teacher Burnout Scale for stress or emotional exhaustion when controlling for individual characteristics.

#### Individual characteristics

##### Teacher self-efficacy (for online instruction and digital media)

Five studies examined the role played in burnout by teacher self-efficacy (Sokal et al., 2020a; Weißenfels et al., 2021), teacher self-efficacy for online instruction (Panisoara et al., 2020; Ma et al., 2021), or self-efficacy for using digital media (Amri et al., 2020; Weißenfels et al., 2021). The results showed that lower teacher self-efficacy (for online instruction) was associated with higher lack of accomplishment and higher emotional exhaustion [three out of three studies; the fourth and fifth study (Amri et al., 2020; Panisoara et al., 2020) reported similar findings using an overall burnout score without distinguishing between the three dimensions]. Higher self-efficacy for using digital media was associated with less change in lack of accomplishment, but not with change in emotional exhaustion (in one out of one study). Relations with depersonalization were less consistent.

##### Attitudes toward, and anxiety around, technology

Two studies examined the relationship between attitudes toward technology and e-learning and burnout (Sokal et al., 2020a; Weißenfels et al., 2021) indicating negative cross-sectional associations, but no associations with change in the three burnout dimensions. One study showed that higher levels of burnout were associated with a lower intention to keep on using online teaching tools in the future and with high extrinsic and low intrinsic motivation for online teaching (Panisoara et al., 2020). Anxiety of using technology and providing virtual instruction was the focus of one study that indicated a positive association with one of the two subscales of the Teacher Burnout Scale, namely stress (Pressley, 2021).

##### Attitudes toward change and adaptability

Two studies examined the association between teacher burnout and teachers' attitudes toward change (Sokal et al., 2020a) and adaptability (Ma et al., 2021). The findings in both studies indicate that higher levels of teacher burnout are associated with less favorable attitudes toward change and adaptability.

##### Personality

Five studies researched the associations between teachers' Big Five personality traits (Collie, 2021), general self-efficacy (Rabaglietti et al., 2021), sense of control (Zhou and Yao, 2020), resilience (Sokal et al., 2020a; Liu et al., 2021), and teacher stress and burnout. These studies showed that higher levels of teacher burnout and stress were associated with higher neuroticism, lower general self-efficacy, sense of control, and resilience, while there was no significant association with extraversion. Greater openness was associated with greater stress, but not with emotional exhaustion (Collie, 2021).^3^

##### Fear or self-rated risk of COVID-19 infection

Three studies studied associations between teacher stress or burnout and teachers' fear of COVID-19 (Carreon et al., 2021; Pressley, 2021) and self-rated risk of getting infected with COVID-19 (Oducado et al., 2021). Findings indicate that higher stress or burnout is associated with a higher level of fear and self-rated risk of getting infected.

##### Social support and basic psychological needs

Three studies examined the role of social support (Amri et al., 2020; Sokal et al., 2020b; Zhou and Yao, 2020). Social support was not associated with stress in one study (Zhou and Yao, 2020), but with burnout in two studies (Amri et al., 2020; Sokal et al., 2020b). One of the latter studies indicated that teachers with higher social support from family and friends experienced higher emotional exhaustion, but greater accomplishment (*r* ≤ 0.15; Sokal et al., 2020b), while the other study indicated that teachers with more social support experienced less symptoms of burnout (Amri et al., 2020). Although Zhou and Yao (2020) did not find a direct association, they showed that higher social support was indirectly associated with lower teacher stress due to a better fulfillment of teachers' basic psychological needs, i.e., higher autonomy, competence, and relatedness.

##### Teaching experience, age, and gender

Six studies examined the role of demographic characteristics in teacher stress and burnout [teaching experience: k = 4 (Amri et al., 2020; Carreon et al., 2021; Collie, 2021; Pressley, 2021); age: k = 3 (Amri et al., 2020; Carreon et al., 2021; Oducado et al., 2021); gender: k= 4 (Amri et al., 2020; Carreon et al., 2021; Collie, 2021; Oducado et al., 2021)]. Older and more experienced teachers had higher burnout scores in two studies, while in the other three studies age and teaching experience were not associated with stress and burnout. Studies were inconclusive regarding the role of gender in stress and burnout.

##### Turnover intention

One study examined the association of burnout and turnover intention, i.e., teachers' intention to quit teaching (Liu et al., 2021). In this study, all three burnout dimensions were associated with higher turnover intention.

##### Self-care activities and stress reduction programs

One study examined associations between teacher burnout and self-care activities indicating that mindfulness, healthy eating, and exercise were associated with higher accomplishment, while associations with other burnout dimensions were negligible (Sokal et al., 2020b). Two intervention studies examined the effects of an inquiry-based stress reduction program (Zadok-Gurman et al., 2021; T1: prior to the pandemic; T2: during the pandemic) and of a program combining stress management strategies and training in technology use for teaching (Pozo-Rico et al., 2020; T1 and T2: during the pandemic). The combined program showed positive effects, indicating a decrease in stress, emotional exhaustion, and depersonalization, and an increase in personal accomplishment for the intervention group, while there were no changes in the control group (Pozo-Rico et al., 2020). The inquiry-based stress reduction program showed no differential effects in the intervention group and control group in terms of stress and personal accomplishment (Zadok-Gurman et al., 2021). There was a differential effect in emotional exhaustion, indicating a smaller increase in the intervention group than in the control group (Zadok-Gurman et al., 2021).

## Discussion

The present study sought to provide a systematic overview of the research into stress and burnout among K−12 teachers during the COVID-19 pandemic. We focused on studies that compared the level of stress and burnout teachers experienced before vs. during the COVID-19 pandemic. We also included studies investigating differences in the levels of stress and burnout experienced by K−12 teachers as compared to individuals employed in other occupational fields. In addition to this, we aimed to identify job and organizational characteristics associated with teacher stress and burnout, but also individual characteristics and activities potentially related to stress and burnout during the COVID-19 pandemic. Based on a systematic literature search, we identified 17 studies examining stress and burnout in 9,874 K−12 teachers.

As to the question of whether K−12 teachers' stress and burnout increased during the COVID-19 pandemic, only two studies out of 17 reported findings on the extent to which teachers' experienced burnout both before and during the COVID-19 pandemic (Weißenfels et al., 2021; control group in Zadok-Gurman et al., 2021). One study found evidence for an increase in lack of accomplishment and depersonalization, but no change in emotional exhaustion (Weißenfels et al., 2021; T2: May 2020; Germany). Another study indicated the reverse: that emotional exhaustion increased, but personal accomplishment and stress did not change (control group in Zadok-Gurman et al., 2021; T2: May 2020; Israel). One reason for these different findings in the two studies could be the different demands and resources in the two countries or samples under investigation, i.e., German teachers vs. Israeli teachers. From a theoretical point of view, the findings by Zadok-Gurman et al. (2021) are in line with the job demands-resources model (Demerouti et al., 2001), which posits that, of the three burnout dimensions, emotional exhaustion develops first, while depersonalization and lack of accomplishment evolve later on. On the other hand, emotional exhaustion is seen as a consequence of work overload, while reduced personal accomplishment is thought to develop when there is a lack of resources (Maslach et al., 2001).

Weißenfels et al. (2021) argue that work overload may not have been the key factor in teachers' experience of the COVID-19 pandemic. Instead, they claim, teachers were lacking information—especially at the start of the pandemic (Kim and Asbury, 2020)—which may have led to reduced levels of personal accomplishment, with teachers creating an emotional distance from their work (Weißenfels et al., 2021). It may also be the case that, at the start of the pandemic, teachers may have activated all of their resources to successfully cope with remote teaching, and the negative consequences of greater emotional exhaustion may have only emerged later on (Kim and Asbury, 2020). Last but not least, while remote teaching posed a number of challenges, some teachers may have had a positive experience of more flexible working—and in some respect less strain—finding that they could work effectively from home (Kim and Asbury, 2020).

On the question of whether K−12 teachers have experienced higher levels of stress and burnout during the COVID-19 pandemic than individuals employed in other occupational fields, two studies found almost no differences between stress levels experienced by K−12 teachers in comparison to teachers in preschools and universities (Ozamiz-Etxebarria et al., 2021) and in comparison to managers, executive employees, and other practitioners (Mari et al., 2021). The only difference that emerged was that K−12 teachers' scores on the PSS-subscale of helplessness were higher than those reported by managers.

When interpreting these results, we need to take into account the time of measurement and regional differences. Ozamiz-Etxebarria et al. (2021) conducted their study when schools and universities had already reopened. The study presumably did not capture potential differences in the stress levels of teachers in different sectors that may have emerged in response to online teaching. The results of Mari et al. (2021) may be limited by the fact that the number of teachers from Southern Italy—which was less affected by the pandemic than Northern Italy—was disproportionately high. The authors did not control for these regional differences and could therefore have underestimated K−12 teachers' actual levels of stress. There were no studies examining differences in burnout levels of K−12 teachers and individuals employed in other occupational fields.

Which job and organizational characteristics relate to K−12 teachers' levels of stress and burnout during the COVID-19 pandemic? Results indicate that school principals' leadership practices are closely associated with teachers' emotional exhaustion (Collie, 2021). Autonomy-thwarting practices, comprising pressure and controlling behaviors, were associated with higher levels of emotional exhaustion in teachers; the association was large in size (ß = 0.46 in a structural equation model, controlling for workload, teachers' personality characteristics, and demographics; Collie, 2021). In contrast, supportive practices, comprising empowerment and understanding, fostered workplace buoyancy or the ability to deal with challenges at work. Workplace buoyancy, in turn, contributed to lower levels of stress and burnout (Collie, 2021). In another study, Sokal et al. (2020b) examined various job demands and resources. Moderate associations only emerged between K−12 teachers' emotional exhaustion and time management, balancing home life and teaching, as well as technology issues (all other bivariate correlations were below 0.30; see also Rabaglietti et al., 2021 for similar findings). Reduced work and the amount of remote teaching were only negligibly related to teachers' stress and burnout (Amri et al., 2020; Collie, 2021). Teachers working in rural as compared to urban or suburban schools experienced similar levels of stress and burnout (Collie, 2021; Pressley, 2021). Taken together, the studies show that having to quickly prepare materials for online teaching while working from home and managing childcare responsibilities were relevant sources of emotional exhaustion (Sokal et al., 2020b). One way school principals can thus support teachers is by avoiding demanding practices and providing a supportive school climate (Collie, 2021).

We now come to the question of which individual characteristics and activities relate to K−12 teachers' levels of stress and burnout during the COVID-19 pandemic. The findings show that teacher self-efficacy in online learning environments were closely associated with teachers' emotional exhaustion and lack of accomplishment (Panisoara et al., 2020; Sokal et al., 2020a; Ma et al., 2021; Weißenfels et al., 2021).^4^ Beyond cross-sectional findings, teachers experienced a lower increase in these dimensions of burnout during the pandemic when their teaching self-efficacy showed a greater increase (Weißenfels et al., 2021). In a similar vein, higher self-efficacy in using digital media was accompanied by a smaller increase in lack of accomplishment, although it was not associated with a change in emotional exhaustion (see also Amri et al., 2020; Pressley, 2021; Weißenfels et al., 2021). This is in line with previous research indicating that teachers who perceive the classroom as more controllable will use better instructional strategies, have favorable teaching experiences, and experience less stress and burnout (Dicke et al., 2014). Given that many teachers had to acquire skills in remote teaching practically overnight (OECD, 2020b; Reimers and Schleicher, 2020), the relevance of teacher self-efficacy in online learning for teacher stress and burnout is comprehensible. In contrast, there was no evidence that attitudes toward e-learning were associated with changes in burnout (Weißenfels et al., 2021; see also mixed evidence in Pressley, 2021; but Amri et al., 2020). Thus, negative attitudes toward e-learning may not make teachers as susceptible to burnout as low teacher self-efficacy in online teaching.

Cross-sectional evidence indicated that teachers were less likely to experience burnout during the COVID-19 pandemic when they had more favorable attitudes toward change and adaptability (Sokal et al., 2020a; Ma et al., 2021), higher general self-efficacy, emotional stability, sense of control, and resilience (Sokal et al., 2020a; Zhou and Yao, 2020; Collie, 2021; Liu et al., 2021; Rabaglietti et al., 2021). These findings are consistent with meta-analyses conducted prior to the pandemic indicating that teachers' personalities—especially a high level of emotional stability—makes them less vulnerable to burnout (Cramer and Binder, 2015; Kim et al., 2019). Although it may seem likely that social support would make teachers less susceptible to burnout, especially during the COVID-19 pandemic, findings on the role of social support for teacher stress and burnout were contentious (Amri et al., 2020; Sokal et al., 2020b; Zhou and Yao, 2020). Previous research has already shown that the link between social support and burnout is weak (e.g., meta-analysis by Halbesleben, 2006). Work-related support, for instance, has been found to be more crucial in reducing burnout than non-work-related support (Halbesleben, 2006; Fiorilli et al., 2019). Studies therefore need to operationalize social support in a clear manner in order to disclose the links between social support and burnout.

Teaching experience, age, and gender were not consistently associated with teacher stress and burnout (Amri et al., 2020; Carreon et al., 2021; Collie, 2021; Oducado et al., 2021; Pressley, 2021). Differences in the strength of these associations could be explained by the time of data collection and the different countries in which the studies were conducted. Teachers who felt more at risk of getting infected with COVID-19 experienced higher levels of stress and burnout (Carreon et al., 2021; Oducado et al., 2021; Pressley, 2021). Thus, teachers living in areas that were more affected by the pandemic or teachers with health issues were more likely to suffer from stress and burnout during this period. Finally, findings indicated that teachers experiencing higher levels of burnout had a higher intention of quitting teaching (Liu et al., 2021). Turnover intentions may have been a result of burnout, but may also have existed prior to the pandemic thus impeding teachers' ability to adapt to remote teaching.

One study indicated that self-care activities, such as mindfulness, healthy eating, and exercise, can be helpful in maintaining personal accomplishment, but the associations were small (Sokal et al., 2020b). The intervention studies indicate that a program combining stress management and training in technology use can effectively reduce stress and burnout (Pozo-Rico et al., 2020), while stress management training alone may not be sufficient (Zadok-Gurman et al., 2021). Although Zadok-Gurman et al. (2021) found that emotional exhaustion increased to a lesser extent in the intervention group than in the control group, the effect was most likely due to higher starting values in the intervention group, which they did not control for in their analyses. While these findings are in line with previous research showing that training in instructional strategies may be more effective than stress management training (e.g., Dicke et al., 2015a), they need to be interpreted cautiously as stress management training programs differ in their effectiveness (Kröll et al., 2017), studies were conducted in different countries (Spain vs. Israel) and the time of measurement differed in both studies (baseline prior to the pandemic in Zadok-Gurman et al., 2021; baseline during the pandemic in Pozo-Rico et al., 2020). Nevertheless, Pozo-Rico et al. (2020) results are in line with research demonstrating that teachers with high teaching self-efficacy in online learning environments experience less stress and burnout (Panisoara et al., 2020; Sokal et al., 2020a; Ma et al., 2021; Weißenfels et al., 2021).

### Limitations and implications for future research

In terms of the measures used, we found that some studies condensed and adapted self-report measures of teacher stress and burnout in order to, for instance, assess the stress that teachers experienced when using technology for online teaching (e.g., Panisoara et al., 2020; Carreon et al., 2021; Ma et al., 2021). Future studies should put more effort into validating these measures. In addition, 14 out of 17 of the studies used cross-sectional designs, and all studies relied on self-report measures. The lack of longitudinal studies is consistent with the review on teacher burnout by Madigan and Kim (2021), who found that only four out of 14 studies—conducted prior to the COVID-19 pandemic—applied a longitudinal study design. Although baseline measures may not be available, because the pandemic could not be foreseen, prospective research could shed light on the longitudinal relationship between teacher stress and burnout and job and organizational characteristics, as well as individual teacher characteristics during the ongoing pandemic.

Future studies should aim to complement ratings of teacher self-efficacy with class-level aggregated ratings of instructional quality that represent a shared perspective from all students in the classroom (Lüdtke et al., 2009). When examining job and organizational characteristics, school-level-aggregated ratings of teaching staff could be insightful (e.g., Kalinowski et al., 2022). In line with the job demands-resources model (Demerouti et al., 2001), buffering effects of job resources moderating the negative consequences of demands should also be examined. Moreover, although some of the studies included in this review assessed both individual and organizational characteristics (e.g., Sokal et al., 2020b; Collie, 2021), they did not aim to identify the interplay of these characteristics. This kind of research design could help answer the question of “‘who' gets burned out in ‘which' situations” during a global pandemic (Chang, 2009, p. 201). While our review provides a systematic overview on individual and organization characteristics that may be relevant in the development of teacher stress and burnout during the pandemic, it lacks quantitative statistical tests. To test for publication bias and more precisely describe mean effect sizes meta-analyses may be insightful.

### Practical implications and conclusion

The present systematic review is based on 17 studies examining stress and burnout in 9,874 K−12 teachers from 20 countries. Most studies focus on the role of individual teacher characteristics for teacher stress and burnout. Studies imply that K−12 teachers' personality, teacher self-efficacy in online teaching, and feeling vulnerable to COVID-19 have been crucial factors in stress and burnout among teachers during the pandemic. On the organizational level, there is some indication that when school principals contribute to a supportive school climate and avoid demanding practices, teachers experience less stress and burnout. In addition, interventions for teachers may potentially be most effective in reducing stress and burnout when they combine stress management and training in technology use. These findings can be seen as important hypotheses that need to be thoroughly examined in intervention studies, using randomized-control designs. Taken together, school principals' leadership coupled with teacher training—aimed at improving stress management and teachers' self-efficacy in online teaching—could help decrease teacher stress and burnout during the ongoing COVID-19 pandemic.

## Data availability statement

The original contributions presented in the study are included in the article/supplementary material, further inquiries can be directed to the corresponding author.

## Author contributions

AW designed and directed the project, conducted the literature search, and took the lead in writing the manuscript. EK was involved in planning the work. AW and EK decided upon inclusion of the initial studies. CH and AW rated the quality of the studies. CH prepared the tables. EK, CH, and MV provided critical feedback and helped shape the manuscript. All authors contributed to the article and approved the submitted version.

## Funding

Funded by the Deutsche Forschungsgemeinschaft (DFG, German Research Foundation) - Projekt nummer 491466077.

## Conflict of interest

The authors declare that the research was conducted in the absence of any commercial or financial relationships that could be construed as a potential conflict of interest.

## Publisher's note

All claims expressed in this article are solely those of the authors and do not necessarily represent those of their affiliated organizations, or those of the publisher, the editors and the reviewers. Any product that may be evaluated in this article, or claim that may be made by its manufacturer, is not guaranteed or endorsed by the publisher.

## Footnotes

^1^Cohen's *d* was calculated for the present review based on the descriptives reported by Zadok-Gurman et al. (2021). Correlation between pre- and post-test was not reported. Following the procedure developed by Fukkink and Lont (2007), we used an estimated correlation of *r* = 0.5.

^2^Cohen's *d* was calculated for the present review based on the descriptives reported by Mari et al. (2021).

^3^Agreeableness and conscientiousness were combined into one latent factor, which the authors called “constraint,” and which was positively associated with greater stress and emotional exhaustion, when controlling for the other personality traits, although, the bivariate correlation with emotional exhaustion was negligible.

^4^Even though two of the studies (Sokal et al., 2020a; Weißenfels et al., 2021) surveyed teachers in terms of their self-efficacy without focusing on online learning environments, teachers supposedly reported on their self-efficacy in these online environments (as the measurement took place while teachers were teaching remotely; for a similar suggestion see Weißenfels et al., 2021).
